# Eigenspace-Based Generalized Sidelobe Canceler Beamforming Applied to Medical Ultrasound Imaging

**DOI:** 10.3390/s16081192

**Published:** 2016-07-28

**Authors:** Jiake Li, Xiaodong Chen, Yi Wang, Wei Li, Daoyin Yu

**Affiliations:** School of Precision Instrument & Opto-electronics Engineering, Tianjin University, Key Laboratory of Opto-electronics Information Technology (Tianjin University), Ministry of Education, Tianjin 300072, China; lijiake1223@tju.edu.cn (J.L.); koala_wy@tju.edu.cn (Y.W.); lycanleeway@163.com (W.L.); dyyu@tju.edu.cn (D.Y.)

**Keywords:** EBGSC, adaptive beamforming, minimum variance, unconstrained optimization beamformer

## Abstract

The use of a generalized sidelobe canceler (GSC) can significantly improve the lateral resolution of medical ultrasound systems, but the contrast improvement isn’t satisfactory. Thus a new Eigenspace-based generalized sidelobe canceler (EBGSC) approach is proposed for medical ultrasound imaging, which can improve both the lateral resolution and contrast of the system. The weight vector of the EBGSC is obtained by projecting the GSC weight vector onto a vector subspace constructed from the eigenstructure of the covariance matrix, and using the new weight vector instead of the GSC ones leads to reduced sidelobe level and improved contrast. Simulated and experimental data are used to evaluate the performance of the proposed method. The Field II software is applied to obtain the simulated echo data of scattering points and circular cysts. Imaging of scattering points show that EBGSC has the same full width at half maximum (FWHM) as GSC, while the lateral resolution improves by 35.3% and 52.7% compared with synthetic aperture (SA) and delay-and-sum (DS), respectively. Compared with GSC, SA and DS, EBGSC improves the peak sidelobe level (PSL) by 23.55, 33.11 and 50.38 dB, respectively. Also the cyst contrast increase by EBGSC was calculated as 16.77, 12.43 and 26.73 dB, when compared with GSC, SA and DS, respectively. Finally, an experiment is conducted on the basis of the complete echo data collected by a medical ultrasonic imaging system. Results show that the proposed method can produce better lateral resolution and contrast than non-adaptive beamformers.

## 1. Introduction

Medical ultrasound imaging, with its characteristics of high transmission capacity and low harm to the human body has become one of the major medical diagnostic technologies nowadays, and imaging algorithms are the key technology of medical ultrasonic imaging [1]. Delay-and-sum (DS) is the most widely used imaging method, but DS suffers from low signal-noise-ratio (SNR) and low resolution [2]. In order to improve the SNR and lateral resolution of echo imaging Jensen [3] proposed synthetic aperture (SA) beamforming. Although SA can equivalently achieve focusing during transmission and reception for every point at the same time in the whole field, and additionally it improves the SNR and lateral resolution of the system, it still has a high level of sidelobe energy. Applying apodization calculations can reduce the sidelobe level at the cost of loss of lateral resolution.

For decades, adaptive beamformers have been used in other array signal processing fields, e.g. antenna and radar, which calculate a weight vector from collected echo data to process the collected echo data. The calculated weight vector is equivalent to a spatial filter which can maintain the desired signal and suppress interference and noise signals of echo data, thus improving the quality of echo imaging. For medical ultrasonic imaging, adaptive beamforming was first introduced by Capon [4]. Synnevåg et al. studied the minimum variance algorithm [5], and Holfort [6] proposed a frequency domain-based minimum variance method. In order to improve the stability of adaptive beamforming, Synnevåg [7] put forward diagonal loading to ultrasonic imaging, and Evans [8] proposed a subaperture averaging algorithm to solve the coherence of ultrasound echoes.

Recently, due to the fact that the generalized sidelobe canceler (GSC) can separate the linear constraints with an adaptive filter, therefore, the constrained optimization problem of adaptive beamforming is converted into an unconstrained optimization problem, which can be further studied by researchers [9,10,11]. A review of the literature about the GSC in ultrasound imaging, shows that a considerable improvement in terms of lateral resolution has been achieved because of the higher resolution of the GSC, but a corresponding improvement in the contrast has not yet been achieved, thus how to improve the contrast of medical ultrasound imaging is a key research direction.

In this paper, we put forward a new eigenspace-based generalized sidelobe canceler (EBGSC) approach for medical ultrasound imaging, which uses the covariance matrix of echo data to construct a signal subspace and a noise subspace to distinguish the desired signal, interference and noise signals of echo data, and by projecting the GSC weight vector onto the constructed signal subspace, the interference and noise signals can be further eliminated. According to scattering points and circular cyst experiments, it is revealed that EBGSC can improve both the lateral resolution and contrast of medical ultrasound systems.

The outline of this paper is as follows: in Section 2 we introduce the sensor signal model, principle of GSC and its application to ultrasound imaging. Section 3 introduces in details the principle and realization of EBGSC. Section 4 presents the experiment results based on simulation data and real echo data. Finally, the advantages of the proposed methodology and its comparison with early proposed adaptive beamformers and non-adaptive beamformers are discussed in Section 5. The whole study is concluded in Section 6.

## 2. Background

### 2.1. Sensor Signal Model

For traditional medical ultrasonic imaging, adaptive beamformers will process the echo data which achieve focusing during transmitting and receiving [3]. For a linear array of M elements, the output of a beamformer is given by [12]:

(1) $$y\left(k\right)=w^{H}\left(k\right)X\left(k\right)=\overset{M}{\underset{i=1}{\sum }}w_{i}^{*}\left(k\right)x_{i}\left(k\right)$$

where k is the time index. X(k) is the time-delayed version of array observations, $X\left(k\right)={\left[x_{1}\left(k\right),x_{2}\left(k\right),\cdots ,x_{M}\left(k\right)\right]}^{T}$, $w\left(k\right)={\left[w_{1}\left(k\right),w_{2}\left(k\right),\cdots ,w_{M}\left(k\right)\right]}^{T}$ is a complex vector of beamforming weights. ${\left(\cdot \right)}^{T}$ and ${\left(\cdot \right)}^{H}$ denote the transpose and conjugate transpose, and ${\left(\cdot \right)}^{*}$ denotes the conjugate complex, respectively. For adaptive beamformers X(k) can be divided into two parts:

(2) X(k)=S(k)+P(k)

where S(k) represents the echo signal of the detected point, which is called desired signal, and P(k) is the interference and noise signals, which include the sidelobe signal, thermal noise and reflection noise, etc.

For adaptive beamformers the weight vector w(k) is calculated from X(k), and varies with the echo data. An ideal weight vector w(k) corresponds to a spatial filter, which can maintain the desired signal while suppressing the interference and noise signals of the echo data. Consequently it enhances the image quality of the medical ultrasound system.

Similarly, Equation (1) can also apply to non-adaptive beamformers. $\forall x_{i}\left(k\right)$ is the receiving focused echo signal of a detected point, and the procedure of receiving focus is also known as delay-and-sum (DS). Setting $w\left(k\right)={\left[1,1,\cdots ,1\right]}^{T}$, the output of the beamformer y(k) is the result of the synthetic aperture (SA), and using a fixed window function like Hamming, Hanning, etc., replaces the weight vector w(k), so the beamforming response will be the result after apodization calculations.

### 2.2. Generalized Sidelobe Canceler (GSC)

The generalized sidelobe canceler (GSC) was originally proposed by Griffiths [13]. GSC can be expressed as constraint conditions, which can maintain the desired signal and suppress the interference and noise signal of echo data:

(3) $$\min w^{H}Rw,\text{ sbuject to }w^{H}a=1$$

where a is the steering vector, it represents the direction of the desired signal, and for transmitting and receiving focused echo data a is a unit vector. R is the interference and noise covariance matrix. The solution of Equation (3) is given by Lagrange multipliers method:

(4) $$w_{\text{MV}}=\frac{R^{-1}a}{a^{H}R^{-1}a}$$

GSC can separate the linear constraints with an adaptive filter, so the constrained optimization problem of Equation (3) is converted into an unconstrained optimization problem. GSC will decompose $w_{\text{GSC}}$ into the adaptive power $w_{a}$, the non-adaptive power $w_{q}$, and $w_{q}$ in constraint subspace, while $w_{a}$ is orthogonal to the constraint subspace.

**Figure 1:**
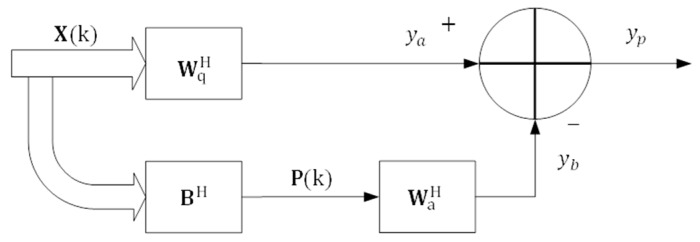
Structure of the generalized sidelobe canceler.

The structure of GSC is shown in Figure 1, where the upper part is called non-adaptive road, where all echo signal received by arrays pass to determine the non-adaptive weight $w_{q}$. In contrast, the lower branch is the adaptive road, which only allows interference and noise signal to pass and determines the adaptive weight $w_{a}$. The weight vector of the system can be expressed as:

(5) $$w_{\text{GSC}}=w_{q}-Bw_{a}$$

(6) $$w_{q}={\left(aa^{H}\right)}^{-1}a$$

(7) $$w_{a}={\left(B^{H}RB\right)}^{-1}B^{H}Rw_{q}$$

where B is a M*(M−1) dimensional blocking matrix, and the role of B is to block off desired signal S(k) and not let it enter the secondary branch: adaptive road [14,15,16], so B must satisfy:

(8) $$B^{H}a=0$$

and in this paper we will set B as:

(9) $$B={\left[\begin{array}{cccccc} 0 & -1 & 0 & 0 & \cdots & 0\\ 0 & 1 & -1 & 0 & \cdots & 0\\ \vdots & \vdots & \vdots & & \vdots & \vdots \\ 0 & 0 & \cdots & 0 & 1 & -1 \end{array}\right]}_{\left(M-1\right)*M}^{T}$$

Observing Equations (5)–(7), the weight vector $w_{\text{GSC}}$ is known when the interference and noise covariance matrix R is obtained. However, it is difficult to obtain an accurate interference and noise covariance matrix R in practical application, thus in practical application the R is replaced by the sample covariance matrix $\hat{R}$, defined by [5]:

(10) $$\hat{R}=\frac{1}{2K+1}\overset{K}{\underset{k=-K}{\sum }}X\left(k\right)X{\left(k\right)}^{H}$$

where 2K + 1 represents the number of echo data used to construct the sample covariance matrix $\hat{R}$, and for adaptive beamforming the 2K + 1 is less than the width of the transmitted ultrasound pulse.

### 2.3. Preprocessing

Ultrasound echo data are broadband and coherent signals, thus GSC can’t be directly applied to process the data [17]. To artificially decorrelate the coherence between echo signals before $\hat{R}$ is calculated, subaperture averaging is used to process the ultrasound echo data. The core of subaperture averaging is to divide the echo data X(k) into P groups of overlapped smoothing submatrix $G_{p}$. The size of $G_{p}$ is L*1, where L≤M/2, and P=M−L+1. And the equation for $\hat{R}$ after subaperture averaging [8] is:

(11) $$\hat{R}=\frac{1}{2K+1}\cdot \frac{1}{P}\overset{K}{\underset{k=-K}{\sum }}\overset{P}{\underset{p=1}{\sum }}G_{p}\left(k\right)G_{p}{\left(k\right)}^{H}$$

and $G_{p}$ is:

(12) $$G_{p}\left(k\right)={\left[x_{p}\left(k\right),x_{p+1}\left(k\right),\cdots ,x_{p+L-1}\left(k\right)\right]}^{T}$$

To improve the stability of the sample covariance matrix $\hat{R}$, another preprocessing algorithm (diagonal loading) is generally used. The core of diagonal loading is to add a noise signal ε into sample covariance matrix $\hat{R}$ and the value of ε is usually small, thereby improves the running stability of algorithm [7]. In this paper we select the ε as:

(13) $$\varepsilon =\frac{1}{\Delta *L}\text{tr}\left\{\hat{R}\right\}$$

where tr{·} is the trace of sample covariance matrix, and the Δ is a fixed number, general varies from 10 to 100.

## 3. Proposed Method

Although GSC can improve the lateral resolution of a medical ultrasonic system, the contrast of image isn’t satisfactory. Especially in a high noise environment, the echo imaging contrast after GSC even slightly worse than using non-adaptive beamformers like SA. We put forward a new eigenspace-based generalized sidelobe canceler (EBGSC) approach for medical ultrasound imaging in this paper, to achieve both high lateral resolution and improved contrast for the echo imaging. Sample covariance matrix $\hat{R}$ is a Hermitian matrix, and Hermitian matrix has the following main properties:

(1) All the eigenvalues of Hermitian matrix are real;
(2) Characteristic vectors of the different characteristic value are orthogonal to each other;
(3) Hermitian matrix $A_{n\times n}$ can be decomposed into $A=E\wedge E^{H}$, and this decomposition is called the spectral theorem. Where $\wedge \;=\text{diag}\left[\lambda_{1},{\text{ λ}}_{2},\cdots ,\lambda_{n}\right]$, $\lambda_{i}$ is the eigenvalue of the matrix **A**. $E=\left[e_{1},e_{2},\cdots ,e_{n}\right]$ is the unitary matrix composed of eigenvectors.

Thus the sample covariance matrix $\hat{R}$ can be decomposed into:

(14) $$\hat{R}=E\wedge E^{H}$$

where $\wedge \;=\text{diag}\left[\lambda_{1},\lambda_{2},\cdots ,\lambda_{L}\right]$, $\lambda_{1}\geq \lambda_{2}\geq \cdots \geq \lambda_{L}$. $E=\left[e_{1},e_{2},\cdots ,e_{L}\right]$. Because of the high coherence of the on-axis signals, the mainlobe-contributed energy concentrates on the eigenvectors associated with the first largest eigenvalue [18,19]. Thus using some larger eigenvalues’ corresponding eigenvectors can construct a signal subspace $E_{s}$, and using the rest of the eigenvalues’ corresponding eigenvectors we can construct the noise subspace $E_{n}$. The signal subspace $E_{s}$ and the noise subspace $E_{n}$ are orthogonal. Therefore by projecting the GSC weight vector $w_{\text{GSC}}$ onto the signal subspace $E_{s}$ one can obtain a new weight vector $w_{\text{EBGSC}}$, and $w_{\text{EBGSC}}$ can maintain the desired signal and further inhibit the interference and noise signal contained in the noise subspace $E_{n}$. The equation for calculating $w_{\text{EBGSC}}$ is:

(15) $$w_{\text{EBGSC}}=E_{s}{E_{s}}^{H}w_{\text{GSC}}$$

Since the mainlobe-contributed energy concentrates on the eigenvectors associated with the first largest eigenvalue, therefore the eigenvectors used to construct the signal subspace $E_{s}$ can be determined by the largest eigenvalue $\lambda_{1}$. The sufficient number of eigenvectors which could effectively retain the mainlobe signal and at the same time could reduce the contribution of undesired sidelobes as much as possible, varies from one point to another point and depends on the relative energy of the mainlobe signal and sidelobe ones [20,21]. Hereby we set a weight coefficient δ, and all the eigenvalues corresponded eigenvectors are used to construct the signal subspace $E_{s}$, if $\lambda_{i}\geq {\delta \lambda }_{1}$.

In order to verify the validity of the proposed algorithm, Field II [22,23] is applied to obtain the echo data of scattering points and circular cysts for simulation experiments. Besides, we also conduct an experiment using the complete echo data which is collected by a medical ultrasonic imaging system. Through a scattering points simulation experiment, we can observe the performance of algorithm in the observation indexes of lateral resolution and the suppression of sidelobe energy. For the circular cyst image, we add a lot of speckle noise around it. By comparing the difference of mean intensity in the cyst region and background we can detect the contrast of each echo image after different beamformers are applied, thereby verifying the validity and superiority of the proposed algorithm.

## 4. Results

In this section, we will provide several examples to compare the performance of the proposed beamformer with GSC, SA and DS in terms of lateral resolution, contrast and sidelobe levels. Field II is a simulation tool widely used in the medical ultrasonic imaging field, and the simulation echo data obtained by Field II can be used as original data to verify the imaging performance of the algorithm. We use Field II to obtain the simulation echo data of scattering points and a circular cyst for a linear phased array ultrasonic system. Key parameters of the simulated linear phased array ultrasonic system are: array number M=64; width of array d=0.2413 mm; center frequency of the ultrasound $f_{0}=3.33\text{ MHz}$; ultrasonic velocity c=1500 m/s; sampling rate of system $f_{s}=71.04\text{ MHz}$. The key parameters of the simulation experiments are same as those of the medical ultrasonic imaging system.

### 4.1. Simulated Point Targets

**Figure 2:**
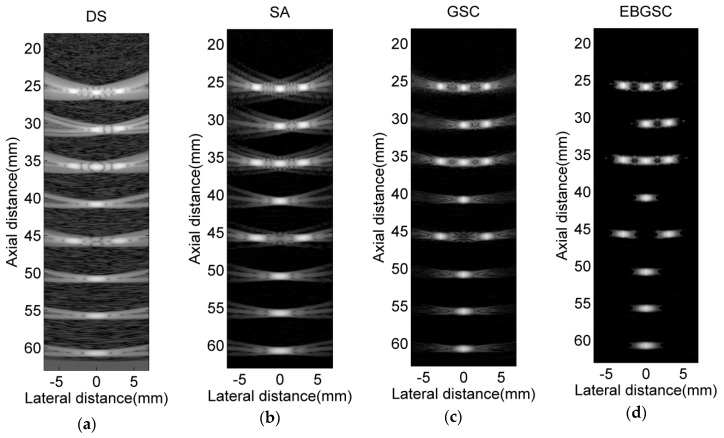
Beamforming responses of 14 point targets using simulated data. (**a**) DS; (**b**) SA; (**c**) GSC (L = 32, K = 0, Δ = 20); (**d**) EBGSC (L = 32, K = 0, Δ = 20, δ = 0.2). And the dynamic range of image is 80 dB.

**Figure 3:**
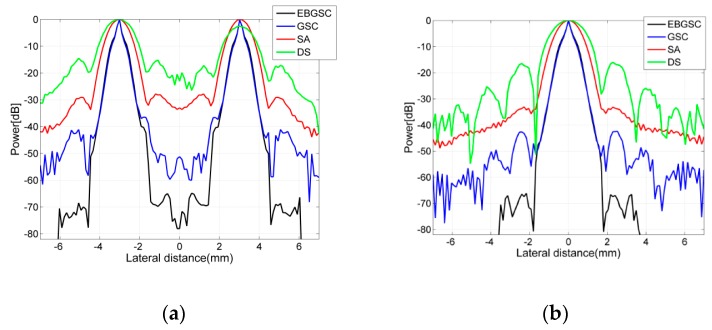
Lateral variation at (**a**) z = 45 mm; (**b**) z = 55 mm of Figure 2a–d.

We set up 14 scattering points in the imaging area. Figure 2 shows the scattering points responses. The beamforming responses of DS are the results of dealing with the delay-and-sum for echo data, in which the 32nd array generates ultrasound and all arrays receive. Figure 3 shows the lateral variation of the beamforming responses at two different depths, and from Figure 3 we can observe the full width at half maximum (FWHM) and the peak sidelobe level (PSL) after each beamformer is used. Taking the depth z = 55 mm as reference, the FWHM and PSL of each algorithm are shown in Table 1. By detecting FWHM, we can see that EBGSC has the same FWHM as GSC, while the FWHM is increased by 35.3% and 52.7% compared with SA and DS, respectively. The observation index PSL is used to check the inhibition of sidelobe energy of each beamformer. For ultrasound echo imaging, the sidelobe energy represents an interference signal, therefore the lower the PSL the better. Comparing each algorithm, with EBGSC the PSL is reduced by 23.55, 33.11, 50.38 dB, respectively, compared to GSC, SA, DS. The scattering points simulation experiment thus shows that EBGSC can improve the lateral resolution and suppress the sidelobe energy.

### 4.2. Circular Cyst Test

**Figure 4:**
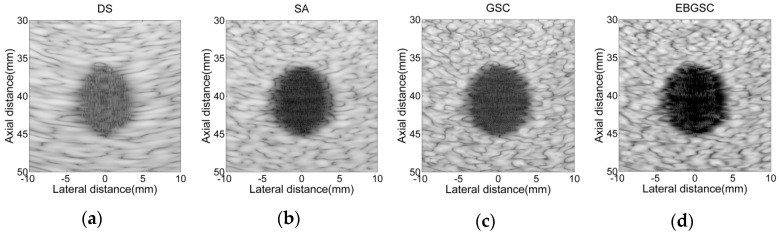
Simulated circular cyst images using Field II. (**a**) DS; (**b**) SA; (**c**) GSC (L = 32, K = 10, Δ = 20); (**d**) EBGSC (L = 32, K = 10, Δ = 20, δ = 0.5). The dynamic range of the image is 80 dB.

**Figure 5:**
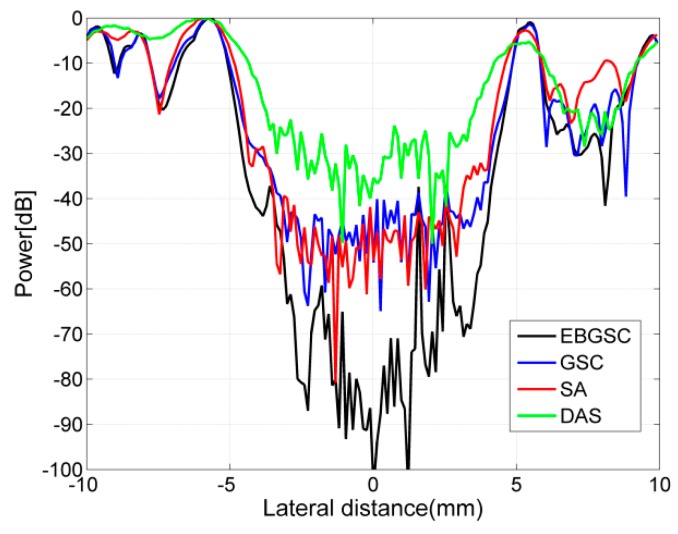
Lateral variation at z = 40 mm of Figure 4a–d.

Based on Field II we obtain a set of echo data for a circular cyst in a high speckle noise environment. The radius of the designed circular cyst is r=5 mm, and its center position is (x,z)=(0,40) mm. We add several scattering points which have different scattering coefficients around the circular cyst, in order to increase the background speckle noise [24]. The beamforming responses after application of the different algorithms are shown in Figure 4. Figure 5 still shows the lateral variation of the beamforming responses, and the contrasts of each algorithm are listed in Table 2, in detail. By comparing the beamforming responses of each algorithm, we can see that EBGSC improves the contrast by 16.77, 12.43, 26.73 dB compared with GSC, SA, DS, respectively. The circular cyst simulation experiment shows that EBGSC has outstanding contrast-enhancing performance for the echo images.

### 4.3. Real Data Experiment

**Figure 6:**
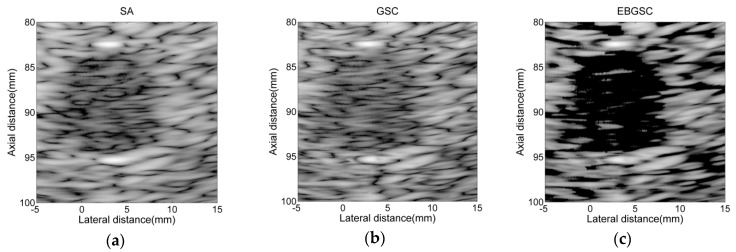
B-mode images of real echo data using (**a**) SA; (**b**) GSC (L = 32, K = 10, Δ = 20); (**c**) EBGSC (L = 32, K = 10, Δ = 20, δ = 0.5). The dynamic range of the image is 40 dB.

**Figure 7:**
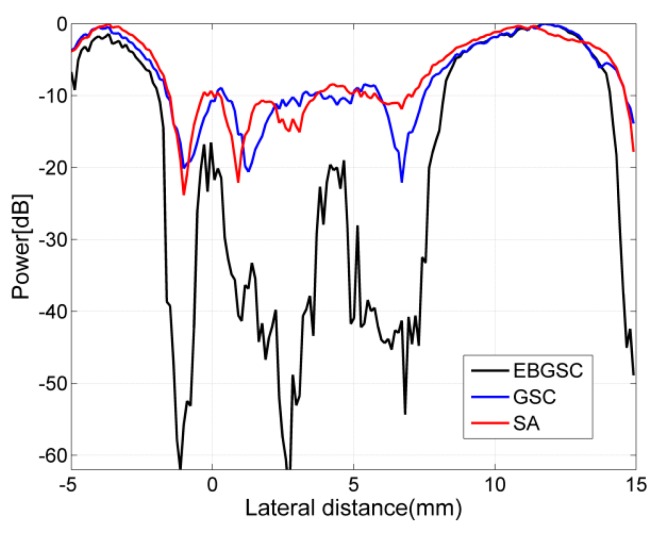
Lateral variation at z = 88 mm of Figure 6a–c.

In order to verify the validity of the algorithm, we also conducted an experiment using the complete echo data collected by a medical ultrasonic imaging system. The beamforming responses after using different algorithms are shown in Figure 6, and Figure 7 shows the lateral variation of the beamforming responses. Compared with the simulated echo data, the echo data collected by the medical ultrasonic imaging system is affected by many factors, such as accuracy of the system parameters, energy of the transmitted ultrasound, and the scattering and attenuation in the tissues, etc. Hence, it has focusing errors and lower signal-to-noise ratio (SNR), therefore we only show the beamforming responses after applying SA, GSC and EBGSC, and the dynamic range of the image is depressed to 40 dB, which is also the dynamic range for medical ultrasonic imaging products in the market. Similarly, we measure the difference of mean intensity in the cyst region and background after applying EBGSC, GSC, and SA, with the contrast values of 30.36, 5.84, and 8.45 dB, respectively. The real data experiment shows that even in a situation of high noise and high focusing errors, EBGSC still has better contrast, and it improves the contrast by 24.52, 21.91 dB in relation to GSC and SA, respectively. The real data experiment results are consistent with the simulation experiments, which validates the effectiveness of the proposed algorithm.

## 5. Discussion

Through scattering points and circular cyst simulation experiments, as well as a real data experiment, we have proved that the proposed EBGSC algorithm can not only increase the lateral resolution, but it also has outstanding performance in enhancing the contrast for ultrasound echo imaging. In the scattering points simulation experiment, we can see that EBGSC has the same lateral resolution as GSC, and adaptive beamformers have obvious advantages in improving system lateral resolution over non-adaptive beamformers. Similarly, adaptive beamformers can more effectively suppress the sidelobe, while improving the PSL.

Likewise, the circular cyst simulation experiment shows that the difference in mean intensity in the cyst region and background after EBGSC drops about 20 dB compared with other beamformers, which validates the effectiveness of EGBSC in improving the contrast for medical ultrasound systems. Comparing beamforming responses after applying GSC and SA, we can see that the contrast of echo imaging after GSC is even slightly below the imaging after using non-adaptive beamformers like SA in the high speckle noise environment, while GSC compared with SA has lower PSL in the scattering points simulation experiment. The reason that gives rise to this curious situation is that the filtering effect of GSC is associated with the degree of freedom of the system, and the degree of freedom of a medical ultrasound system after subaperture averaging is L-1. The adaptive beamforming will have good performance with the condition that the number of interference sources is less than L-2, while its filtering effect will be weakened when the number of interference sources is more than L-2, while the weight vector of EBGSC is obtained by projecting the GSC weight vector onto a vector subspace constructed from the eigenstructure of the sample covariance matrix, which can further eliminate the influence of interference and noise, therefore, it will have more evident advantages in improving contrast for echo images.

**Figure 8:**
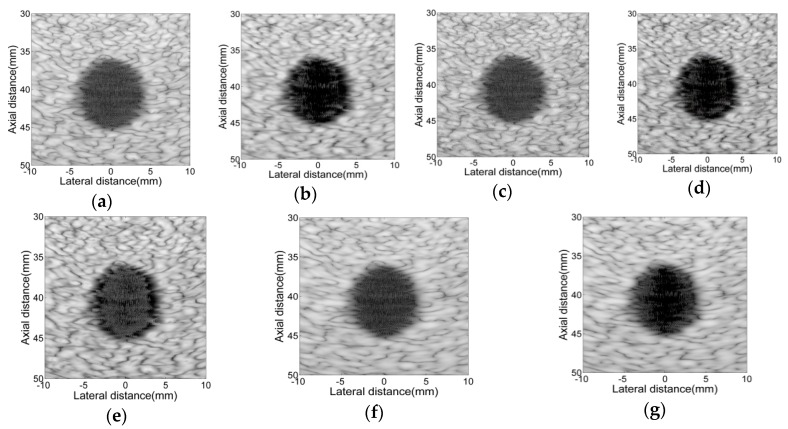
Simulated circular cyst images using Field II. (**a**) GSC (L = 32, K = 20, Δ = 20); (**b**) EBGSC (L = 32, K = 20, Δ = 20, δ = 0.5); (**c**) GSC (L = 32, K = 10, Δ = 100); (**d**) EBGSC (L = 32, K = 10, Δ = 100, δ = 0.5); (**e**) EBGSC (L = 32, K = 10, Δ = 20, δ = 0.1); (**f**) GSC (L = 16, K = 10, Δ = 20); (**g**) EBGSC (L = 16, K = 10, Δ = 20, δ = 0.5). The dynamic range of the image is 80 dB.

**Figure 9:**
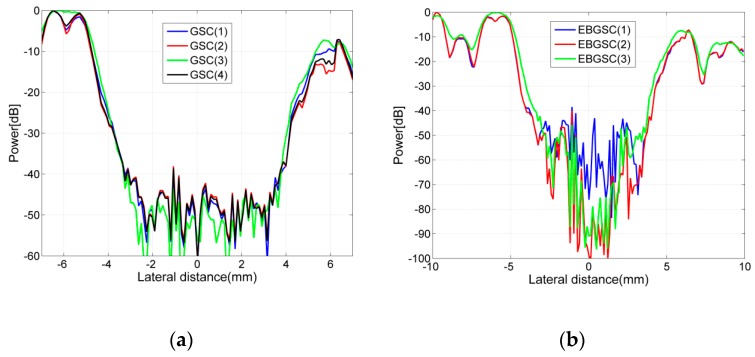
Lateral variation at z = 40 mm of Figure 8. (**a**) GSC (1): L = 32, K = 20, Δ = 20; GSC (2): L = 32, K = 10, Δ = 100; GSC (3): L = 16, K = 10, Δ = 20; GSC (4): L = 32, K = 10, Δ = 20; (**b**) EBGSC (1): L = 32, K = 10, Δ = 20, δ = 0.1; EBGSC (2): L = 32, K = 10, Δ = 20, δ = 0.5; EBGSC (3): L = 16, K = 10, Δ = 20, δ = 0.5.

For adaptive beamformers, we introduce a lot of preprocessing algorithms, and we also set some parameters, hereby we will roughly discuss the impact of these parameters on adaptive beamformers. The beamforming responses of adaptive beamformers using different parameters are shown in Figure 8, and the contrasts of each echo image are listed in Table 3, Figure 9 shows the lateral variation of the beamforming responses. We can see that the parameter L and δ have more noticeable influence on imaging quality. Parameter L determines the degree of freedom of the medical ultrasound system, and when setting L = 1, adaptive beamformers will degenerate into the non-adaptive beamformer (SA) scenario. For a medical ultrasound system, the larger the L is, the better the lateral resolution of the system will be. As can be seen in Figure 9, when choosing L = 16, the beamforming responses of a circular cyst have a smaller size in the lateral direction compared with that when L = 32. The parameter δ determines the number of eigenvectors which are used to construct the signal subspace. By setting a larger δ, more interference and noise signal will be eliminated. As seen in Figure 9b, when choosing δ = 0.1, EBGSC won’t result in an obvious contrast improvement compared with GSC. Parameter K determines the number of echo data used to build the sample covariance matrix. The parameter K has a temporal smoothing effect, which smooths the echo image. Parameter Δ determines the noise energy added to the sample covariance matrix, and the bigger Δ is the smaller the noise energy that will be added, and stability of the sample covariance matrix will be reduced.

## 6. Conclusions

In this paper, we project the GSC weight vector onto a vector subspace constructed from the eigenstructure of the covariance matrix, and by replacing the GSC weight vector with the new proposed one, interference and noise signals can be further reduced, thus the new algorithm can improve both the lateral resolution and contrast of a medical ultrasound system. Through scattering points and circular cyst simulation experiments and a real data experiment we have proved the effectiveness of the new algorithm.

## Figures and Tables

**Table 1:** FWHM and PSL of scattering points at depth z = 55 mm.

Algorithm	FWHM (mm)	PSL (dB)
DS	2.05	−15.82
SA	1.50	−33.08
GSC	0.97	−42.65
EBGSC	0.97	−66.20

**Table 2:** Contrast of beamforming responses for different beamforming algorithms.

Algorithm	Mean Intensity in the Cyst Region (dB)	Mean Intensity in the Background (dB)	Contrast (dB)
DS	−44.61	−20.20	24.41
SA	−59.96	−21.26	38.71
GSC	−57.36	−22.99	34.37
EBGSC	−75.84	−24.70	51.14

Contrast = mean intensity in the background–mean intensity in the cyst region.

**Table 3:** The contrast for adaptive beamforming setting different parameters.

	Parameters	Mean Intensity in the Cyst Region (dB)	Mean Intensity in the Background (dB)	Contrast (dB)
GSC	L = 32, k = 10, Δ = 20	−57.36	−22.99	34.37
GSC	L = 16, k = 10, Δ = 20	−59.84	−22.14	37.70
GSC	L = 32, k = 20, Δ = 20	−56.42	−21.58	34.84
GSC	L = 32, k = 10, Δ = 100	−57.26	−23.87	33.39
EBGSC	L = 32, k = 10, Δ = 20, σ = 0.5	−75.84	−24.70	51.14
EBGSC	L = 16, k = 10, Δ = 20, σ = 0.5	−72.80	−22.81	49.99
EBGSC	L = 32, k = 20, Δ = 20, σ = 0.5	−78.61	−23.32	55.29
EBGSC	L = 32, k = 10, Δ = 100, σ = 0.5	−76.38	−26.12	50.26
EBGSC	L = 32, k = 10, Δ = 20, σ = 0.1	−61.52	−24.70	36.83
